# A high-fat diet has negative effects on tendon resident cells in an in vivo rat model

**DOI:** 10.1007/s00264-022-05340-1

**Published:** 2022-02-24

**Authors:** Scott M. Bolam, Subhajit Konar, Young-Eun Park, Karen E. Callon, Josh Workman, A. Paul Monk, Brendan Coleman, Jillian Cornish, Mark H. Vickers, Jacob T. Munro, David S. Musson

**Affiliations:** 1 Department of Medicine, University of Auckland, 85 Park Road, Grafton, Auckland New Zealand; 2 Department of Orthopedic Surgery, Auckland City Hospital, 2 Park Road, Grafton, Auckland New Zealand; 3 Chemical and Materials Engineering, University of Auckland, 5 Grafton Rd, Auckland, New Zealand; 4 Auckland Bioengineering Institute, University of Auckland, 70 Symonds St, Grafton, Auckland New Zealand; 5 Department of Orthopedic Surgery, Middlemore Hospital, 100 Hospital Road, Otahuhu, Auckland New Zealand; 6 Liggins Institute, University of Auckland, 85 Park Road, Grafton, Auckland New Zealand; 7 Department of Nutrition & Dietetics, University of Auckland, 85 Park Road, Grafton, 1023 Auckland New Zealand

**Keywords:** Obesity, Achilles tendon, High-fat diet, Animal model

## Abstract

**Background:**

Tendinopathy is a major complication of diet-induced obesity. However, the effects of a high-fat diet (HFD) on tendon have not been well characterised. We aimed to determine: [1] the impact of a HFD on tendon properties and gene expression; and [2] whether dietary transition to a control diet (CD) could restore normal tendon health.

**Methods:**

Sprague–Dawley rats were randomised into three groups from weaning and fed either a: CD, HFD or HFD for 12 weeks and then CD thereafter (HF-CD). Biomechanical, histological and structural evaluation of the Achilles tendon was performed at 17 and 27 weeks of age. Tail tenocytes were isolated with growth rate and collagen production determined. Tenocytes and activated THP-1 cells were exposed to conditioned media (CM) of visceral adipose tissue explants, and gene expression was analysed.

**Results:**

There were no differences in the biomechanical, histological or structural tendon properties between groups. However, tenocyte growth and collagen production were increased in the HFD group at 27 weeks. There was lower SOX-9 expression in the HFD and HF-CD groups at 17 weeks and higher expression of collagen-Iα1 and matrix metalloproteinase-13 in the HFD group at 27 weeks. THP-1 cells exposed to adipose tissue CM from animals fed a HFD or HF-CD had lower expression of Il-10 and higher expression of Il-1β.

**Conclusions:**

In this rodent model, a HFD negatively altered tendon cell characteristics. Dietary intervention restored some gene expression changes; however, adipose tissue secretions from the HF-CD group promoted an increased inflammatory state in macrophages. These changes may predispose tendon to injury and adverse events later in life.

**Supplementary Information:**

The online version contains supplementary material available at 10.1007/s00264-022-05340-1.

## Introduction

Obesity has received considerable attention regarding its pathophysiological link to various chronic conditions [1, 2]. There is a strong association between obesity and musculoskeletal disorders including osteoarthritis, osteoporosis and lower back pain [3–5]. More recently, obesity has been identified as a strong risk factor for tendon-related disorders [6–11].

Obesity-related tendon pathology has been observed in elbow, foot, patella and rotator cuff tendons [6]. However, the Achilles tendon appears particularly prone to the deleterious effects of obesity [9–13], with one study demonstrating that people who were overweight or obese were 2.6 and 6.6 times more likely, respectively, to be affected by Achilles tendinopathy than people with a healthy body weight [11].

The impact of obesity on tendon pathology is poorly understood partly because symptoms only present when there is acute injury or severe co-morbidity. Clinical studies do not identify the role of obesity alone, due to confounding variables such as increased age, genetics, obesity duration and the amount of overload the tendons are exposed to. Sprague–Dawley rats fed a high-fat diet (HFD) are a well-established model of diet-induced obesity that increases fat and body mass, and leads to systemic and localised inflammation within musculoskeletal tissues [14, 15].

Recently, studies have demonstrated increased levels of pro-inflammatory cytokines that are secreted by adipose tissue in obese patients [7, 16–21]. In tendinopathy, macrophages migrate into tendon tissue and co-ordinate both the activation and resolution of inflammation [22]. These adipose secretions may play an important role through their effects on tenocytes and macrophages that has not been well characterised.

The aims of this study were: (1) to evaluate the pathogenic link between a high-fat diet (HFD) and poor tendon health; (2) determine whether dietary intervention from a HFD to a control diet (CD) can restore normal tendon health. In order to achieve these aims, we used an established rodent model of diet-induced obesity to assess: (1) tendon tissue properties biomechanically and histologically, (2) cellular changes using isolated primary rat tenocytes, and (3) an in vitro co-incubation model of visceral fat-derived conditioned media (CM) with the tenocytes and the human macrophage cell line, THP-1 [23], to study the potential effects of adipose secretions.

## Methods

All animal experimentation was approved by the University of Auckland Animal Ethics Committee (#002,147) and conformed to the ARRIVE guideline [24].

### Animal model

**Fig. 1:**
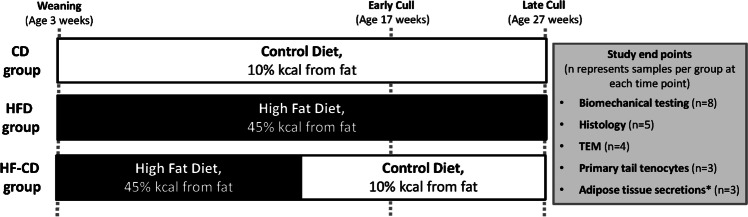
The study design and timeline. TEM: transmission electron microscopy. *samples were collected at late time point only

Seventy-eight Sprague–Dawley male rats were sourced at weaning and randomly allocated into weight-matched groups. Each group was fed ad libitum either: [1] a CD (10% kcal from fat, #D12450H, Research Diets, New Brunswick, NJ, USA) [2] a HFD (45% kcal from fat, #D12451, Research Diets) or [3] a HFD for 12 weeks, then a CD thereafter (HF-CD) (Fig. 1). Animals were housed under standard conditions at 22 °C with a 12 hour light–dark cycle.

All animals in this study underwent left rotator cuff tenotomy and immediate repair at 15 weeks of age as part of a study looking at the effects of obesity on rotator cuff healing [25].

In this study, end points were (1) biomechanical, histological and structural properties of Achilles tendons; (2) tenocyte proliferation, collagen production and gene expression and (3) effects of CM from visceral adipose tissue secretions on tenocytes and THP-1 cells.

At 17 and 27 weeks of age, rats were euthanised by carbon dioxide inhalation. Both Achilles tendon complex were excised either wrapped in saline-soaked gauze and stored at -20 °C for later biomechanical testing; immersed in 10% neutral buffered formalin for histological analysis; or immersed in 2.5% glutaraldehyde for transmission electron microscopy (TEM). The tails were collected, and primary tenocytes were harvested from tendon fascicles. Intra-abdominal fat biopsies were obtained from each group at 27 weeks of age only.

Analytical methods are only briefly stated; however, detailed descriptions are included in the Supplementary Methods.

### Biomechanical testing

In brief, the biomechanical properties of the Achilles tendons were measured using an ElectroPuls E3000 (Instron, Norwood, MA, USA), following an established procedure [26, 27].

### Histological analysis

Briefly, excised Achilles tendon were fixed, decalcified, embedded, cut in coronal sections and then stained with haematoxylin–eosin (H&E). Sections viewed using both transmitted and polarised light. Cell density and nuclear aspect ratio were analysed using the ImageJ software (NIH, Bethesda, MD, USA). The Directionality plug-in for Fiji (http://fiji.sc/Fiji, Ashburn, VA, USA) was used to measure collagen fibre alignment. Six regions of interest were measured for each sample.

### Collagen fibril diameter measurement with TEM

Tendon sections were imaged on a Tecnai G^2^ spirit twin transmission electron microscope (FEI, Hillsboro, OR, USA) at 120 kV with a Morada camera (Olympus Soft Imaging Solution, Munster, Germany). The diameter of each collagen fibril was analysed using the ImageJ software. At least five images were analysed per sample.

### Primary tenocyte cell culture, growth assays and collagen deposition from tail tendon

Primary rat tenocytes were isolated from tail tendon fascicles, as previously described [28]. Tenocytes were seeded and incubated in Dulbecco’s modified Eagle’s Medium: Nutrient Mixture F-12 (DMEM: F-12) with 5% foetal bovine serum (FBS). Tenocyte growth was measured with alamarBlue (ThermoFisher Scientific, Waltham, MA, USA) every 24 hours for a total of 72 hours. Tenocyte collagen deposition was measured using Sirius red after 72 hours. There were four wells per biological repeat.

### Adipose tissue-conditioned media tenocyte culture

Adipose CM was used to study the depot-specific effect of visceral omental fat on rat tenocytes. CM was generated by incubating the adipose tissue explants in a standardised volume of DMEM: F-12 with 1% FBS (200 mg tissue/mL media) for 48 h. Tenocytes obtained from tail tendon of a 17-week-old male rat fed a CD were then cultured in adipose CM and DMEM/F12 with 1% FBS (ratio 3.5:6.5) for 72 hours. Cell growth assays with alamarBlue and collagen deposition assays with Sirius red were then performed, after 48 hours.

### Adipose tissue-conditioned media THP-1 cell culture

An established in vitro co-incubation model was used to study the effects of adipose CM on primary rat tenocytes and THP-1 cells [23, 29]. Briefly, human monocytic THP-1 cells (TIB-202™, ATCC, Manassas, VA, USA) were seeded in Roswell Park Memorial Institute (RPMI) cell media and then activated to macrophage-like cells. After 24 hours, media were replaced with either adipose CM and RPMI with 10% FBS from each group or control media. Plates were incubated for 48 hours post-seeding. Cell pellets were then harvested, washed and stored for gene expression analysis.

### Cytokine profiling in adipose tissue-conditioned media

The concentration of adiponectin and leptin in the adipose CM was analysed by commercial rat-specific enzyme-linked immunosorbent assays (ELISA; Crystal Chem, Chicago, IL, USA). Insulin, interleukin (Il)-1β, Il-6, and tumour necrosis factor α (TNFα) were analysed by Quantikine ELISA (R&D Systems; Minneapolis, MN, USA).

### Gene expression analysis

For analysis of gene expression, total cellular RNA was extracted from cultured cells using the RNeasy minikit (Qiagen, Venlo, The Netherlands). The ΔΔCt calculation method was used to determine relative expression [30], normalised to the values of cells from the tail tendon of a 17-week-old male rat fed a CD.

### Statistical analysis

Data were analysed using either a one-way or a two-way analysis of variance (ANOVA) with post hoc Tukey’s test. *P* < 0.05 was considered significant. Data are presented as mean ± standard error of the mean (SEM) and graphed using Prism 8 software (GraphPad Software, Inc., La Jolla, CA, USA).

## Results

### A HFD did not alter Achilles tendon properties

There was no difference in any biomechanical or histological parameters measured between animals fed a CD, HFD or HF-CD at either 17 or 27 weeks of age (Tables 1 and 2, Supplemental Figs. 1 and 2).

**Table 1:** Biomechanical testing results. Data are presented as mean ± SEM (*n* = 8 per group)

Interaction	Cross-sectional area (cm^2^)			Ultimate load at failure (N)			Young’s modulus (MPa)		
		*CD vs. HFD*	*HFD vs. HF-CD*		*CD vs. HFD*	*HFD vs. HF-CD*		*CD vs. HFD*	*HFD vs. HF-CD*
17 weeks of age									
CD	3.91 ± 0.22	—	—	81.05 ± 4.61	—	—	134.27 ± 13.69	—	—
HFD	4.57 ± 0.34	*P* = 0.3594	—	71.81 ± 4.59	*P* = 0.3733	—	102.03 ± 14.67	*P* = 0.1548	—
HF-CD	5.02 ± 0.25	*P* = 0.0755	*P* = 0.6372	77.72 ± 5.06	*P* = 0.8938	*P* = *0.7027*	103.57 ± 14.62	*P* = *0.2306*	*P* = *0.9961*
27 weeks of age									
CD	4.84 ± 0.46	—	—	71.31 ± 6.76	—	—	110.59 ± 11.75	—	—
HFD	5.61 ± 0.37	*P* = *0.2541*	—	75.82 ± 3.72	*P* = 0.7870	—	82.70 ± 4.26	*P* = *0.2429*	—
HF-CD	4.75 ± 0.34	*P* = *0.9781*	*P* = *0.1797*	81.41 ± 4.33	*P* = 0.3646	*P* = *0.7288*	110.30 ± 14.65	*P* = *0.9999*	*P* = *0.3026*

**Table 2:** Detailed histologic scores. Data are presented as mean ± SEM (*n* = 5 per group)

	Cellularity (cells per mm^2^)			Nuclei circularity (0–1)			Directionality goodness value (0–1)		
Interaction		*CD vs. HFD*	*HFD vs. HF-CD*		*CD vs. HFD*	*HFD vs. HF-CD*		*CD vs. HFD*	*HFD vs. HF-CD*
17 weeks of age									
CD	1163.46 ± 79.97	—	—	0.576 ± 0.036	—	—	0.902 ± 0.020	—	—
HFD	1348.33 ± 67.88	*P* = 0.2294	—	0.614 ± 0.021	*P* = 0.5741	—	0.913 ± 0.018	*P* = 0.8649	—
HF-CD	1275.33 ± 78.04	*P* = 0.5700	*P* = 0.7843	0.601 ± 0.030	*P* = 0.7782	*P* = *0.9392*	0.918 ± 0.015	*P* = 0.7423	*P* = *0.9724*
27 weeks of age									
CD	1057.83 ± 29.23	—	—	0.581 ± 0.010	—	—	0.910 ± 0.012	—	—
HFD	1042.64 ± 53.86	*P* = *0.9894*	—	0.621 ± 0.024	*P* = 0.5403	—	0.932 ± 0.008	*P* = 0.5677	—
HF-CD	998.17 ± 122.62	*P* = *0.8497*	*P* = *0.9132*	0.567 ± 0.029	*P* = 0.9225	*P* = *0.3299*	0.891 ± 0.016	*P* = 0.6563	*P* = *0.1579*

### Tenocytes derived from animals fed a HFD had higher rates of cell proliferation and collagen production, with gene expression changes indicating higher rates of collagenous matrix turnover

**Fig. 2:**
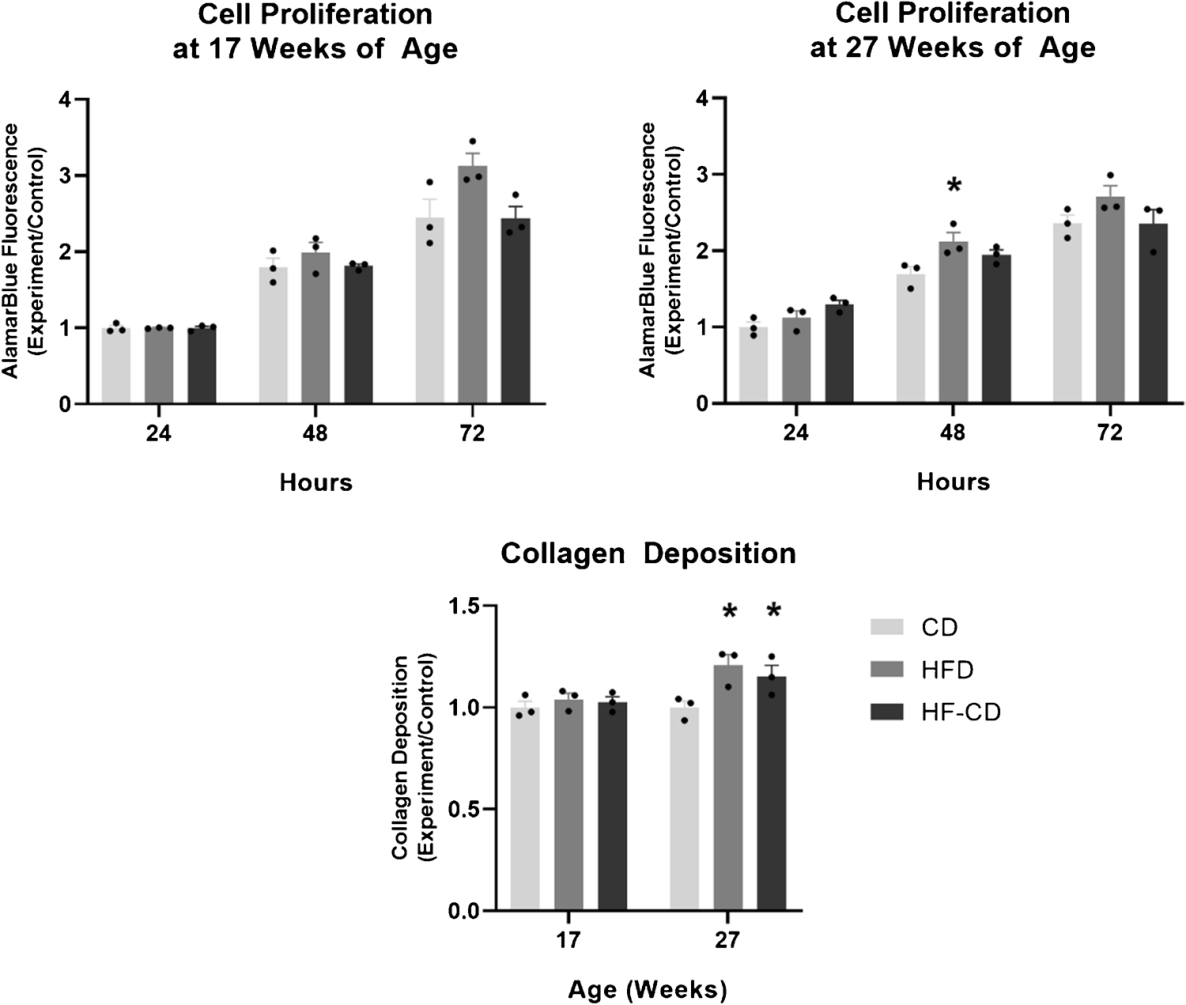
A HFD increased tenocyte cell proliferation at 48 h, as determined by alamarBlue assay and increased primary rat tail tenocyte collagen deposition, as determined by Sirius red assay. * = significantly different from control (*p* < 0.05). Data are presented as mean ratio of control ± SEM (*n* = 3 per group)

Tenocytes derived from 27-week-old animals fed a HFD had a significantly higher proliferation rate (25%, *p* = 0.035) at 48 h compared to animals fed a CD, but there were no significant changes in the HF-CD group. There was significantly higher collagen deposition in the cells derived from 27-week-old animals in the HFD and HF-CD groups (21%, *p* = 0.008 and 15%, *p* = 0.043, respectively) compared to the control group. There was no statistically significant difference between groups at 17 weeks of age (Fig. 2).

**Fig. 3:**
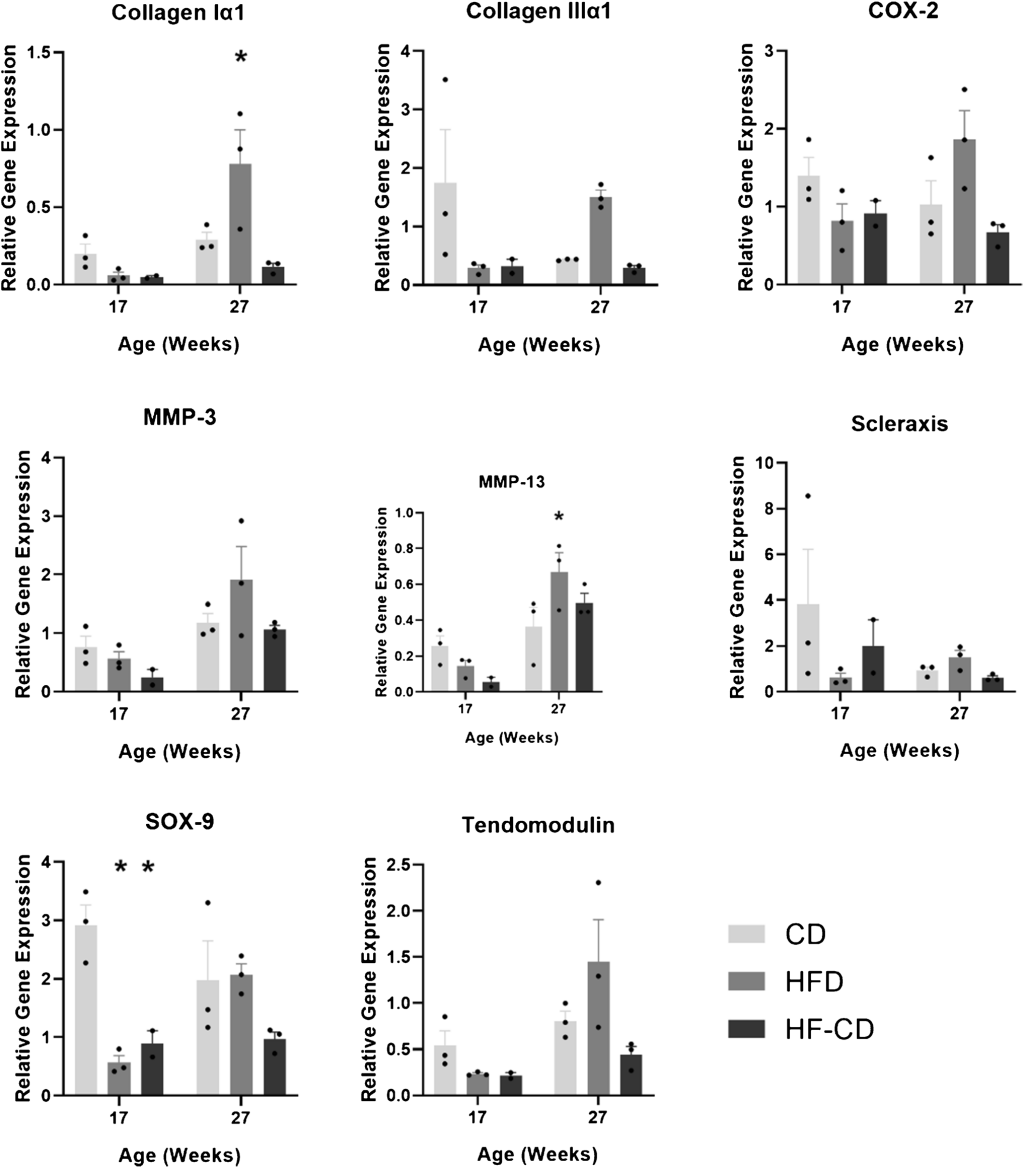
Effect of a HFD on the gene expression profile of primary tenocytes derived from tail tendon. * = significantly different from control (*p* < 0.05). Data are presented as mean ± SEM (*n* = 3 per group)

At 17 weeks of age, tenocyte expression of SRY-box-containing gene-9 (SOX-9) was fivefold (*p* = 0.001) and threefold (*p* = 0.009) lower in the HFD and HF-CD groups, respectively, compared to control group. At 27 weeks of age, collagen Iα1 (COLIα1) and matrix metalloproteinase (MMP)-13 expression were both significantly higher in the HFD compared to control by approximately twofold (*p* = 0.0143 and *p* = 0.0367, respectively). There were no changes in the expression of other tenocytic or osteoblastic gene markers (Fig. 3).

### There was no overt change in the cytokine and adipokine profiles of adipose tissue-conditioned media between groups

There were no differences in the concentrations of cytokines (Il-1β, Il-6 and TNFα) and adipokines (leptin and adiponectin) in adipose tissue CM between dietary groups taken from 27-week-old animals (Table 3).

**Table 3:** Cytokine and adipokine profiles of adipose tissue CM taken from 27-week-old animals. Data are presented as mean ± SEM (*n* = 3 per group)

	27 Weeks of age					
Study group	CD	HFD		HF-CD		
Interaction			*CD vs. HFD*		*CD vs. HF-CD*	*HFD vs* *HF-CD*
Leptin (ng/mL)	4.19 ± 1.43	6.17 ± 3.73	*N.S*	0.93 ± 0.40	*N.S*	*N.S*
Adiponectin (mg/mL)	4.12 ± 1.07	4.55 ± 1.51	*N.S*	4.84 ± 0.32	*N.S*	*N.S*
Insulin (ng/mL)	75.03 ± 0.03	79.67 ± 2.63	*N.S*	75.03 ± 1.10	*N.S*	*N.S*
Il-1β (ρg/mL)	2.36 ± 0.12	2.70 ± 0.11	*N.S*	2.65 ± 0.33	*N.S*	*N.S*
Il-6 (ng/mL)	63.59 ± 3.59	47.36 ± 8.93	*N.S*	50.19 ± 6.36	*N.S*	*N.S*
TNFα (ρg/mL)	0.66 ± 0.05	0.78 ± 0.08	*N.S*	0.70 ± 0.00	*N.S*	*N.S*

### Exposure to adipose tissue-conditioned media of animals fed a HFD led to higher tenocyte proliferation and collagen production. Adipose secretions from the HFD and HF-CD groups induced pro-inflammatory changes in THP-1 cells

**Fig. 4:**
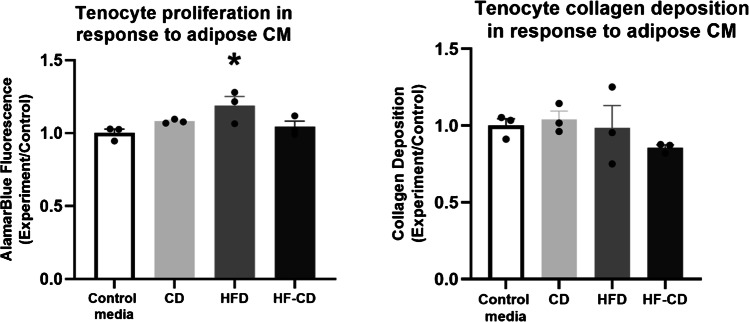
CM from adipose tissue of animals fed a HFD increased tenocyte cell proliferation; however, there was no difference in collagen deposition rate between groups,* = significantly different from control (*P* < 0.05). Data are presented as mean ratio of control media ± SEM (*n* = 3 per group)

**Fig. 5:**
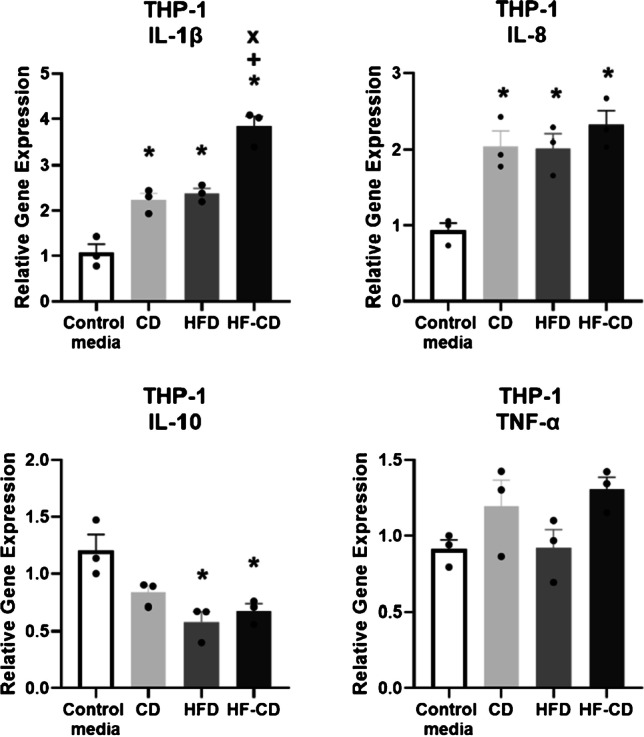
Relative gene expression of three pro-inflammatory markers (Il-1β, TNFα and Il-8) and one anti-inflammatory marker (Il-10) from THP-1 cells culture in CM from adipose tissue from each group. * = *P* < 0.05 vs. control media, +  = *P* < 0.05 vs. CD, x = *P* < 0.05 vs. HFD. Data are presented as mean ratio of control media ± SEM (*n* = 3 per group)

In tenocytes exposed to adipose tissue CM from the HFD group, higher cell proliferation (18%, *p* = 0.043) was observed in comparison with control media. However, there was no difference in collagen deposition in any group (Fig. 4). Compared to control media, THP-1 cells exposed to adipose tissue CM from all rats had two- to threefold higher expression of Il-1β, consistent with a pro-inflammatory response. This was most marked in the HF-CD group, which was twofold higher than the CD (*p* < 0.001) and the HFD (*p* < 0.001) groups. Expression of Il-8, a pro-inflammatory marker, was twofold higher in all groups compared to control media (*p* = 0.007, 0.009 and 0.002, respectively). The expression of Il-10, an anti-inflammatory marker, was twofold lower in THP-1 cells exposed to adipose tissue CM from both the HFD and HF-CD groups, in comparison with control media (*p* = 0.007 and 0.018, respectively). There was no difference in the expression of TNFα between any groups (Fig. 5).

## Discussion

This study provides further evidence that obesity can negatively impact tendon outcomes. A HFD leads to negative changes in tenocyte proliferation, collagen production and gene expression profile. Furthermore, adipose secreted factors from animals fed a HFD increased tenocyte proliferation and promoted a pro-inflammatory gene expression profile in macrophages, that is consistent with changes seen in tendinopathy. Dietary intervention resolved some, but not all of the gene expression changes induced by the HFD. However, adipose tissue secretions from the HF-CD groups also induced an increased pro-inflammatory state in macrophages, which was significantly higher than all other groups. Interestingly, we did not observe any differences in the mechanical and histological properties of the Achilles tendon between the groups.

In this study, tenocytes demonstrated a shift in phenotype with exposure to a HFD. A HFD lowered expression of SOX-9 in tendon cells derived from 17-week-old animals, whereas there were higher expression of COLIα1 and higher expression of MMP-13 in animals at 27 weeks of age. SOX-9 expression is essential in controlling musculoskeletal system development, including muscle, tendon and bone. Decreased SOX-9 expression in tendons is associated with cartilage hypoplasia at its insertion to muscle [31]. Previously work by Grewal et al. [32] observed increased expression of MMPs in the tail tendon of mice fed a HFD. MMPs cause catabolic matrix remodelling with collagenolytic activity, and basal activity of most MMPs is greatly modified in painful tendinopathy [33]. We also observed higher COLIα1 gene expression reflecting increased matrix synthesis and higher collagen deposition rates in the HFD and HF-CD groups compared to controls. These changes indicate a relatively higher collagenous matrix turnover rate in tendon with a HFD.

Interestingly, a HFD did not alter the Achilles tendon biomechanical properties or cross-sectional area after up to 24 weeks of dietary exposure. We also observed no histological alterations or changes in collagen fibril diameter with a HFD. In human clinical studies, higher body mass index (BMI) is associated with macroscopic tendon changes including increased thickness and decreased stiffness [34–36]. A few previous studies have found that a HFD altered the biomechanical properties of rodent tendon [32, 37, 38]. However, the influence of obesity on tendon properties has not been reported consistently [39, 40]. This suggests that although we observed changes in tenocyte gene expression, the detrimental effects of a HFD on tendon structure and biomechanical properties may only be unmasked after a tendon injury or age-related degeneration.

In the previous study using this animal cohort [25], a HFD led to increased body and fat mass, and impaired enthesis healing after rotator cuff repair surgery, with inferior biomechanical and histological outcomes. Restoring normal weight with dietary change after surgery did not improve healing outcomes. Interestingly, circulating leptin concentrations were negatively correlated with load to failure and poor histological structure of the repaired enthesis, suggesting a biochemical link between HFD/obesity and poor tendon outcomes.

Using CM from adipose tissue explants, all the secreted factors from different cells types in adipose tissue are captured that reflects their proportion and relative contribution in vivo [29]*.* Macrophages migrate into tendon tissue during tendon inflammation and then co-ordinate the activation and resolution of tendinopathy [22]. We used THP-1 cells, a monocytic cell line that can be activated into mature macrophages in vitro [23]*,* to investigate the influence of adipose CM on macrophage activation*.* In response to adipose CM from the HFD and HF-CD group, the relative gene expression of anti-inflammatory cytokine Il-10 was lower compared to control media. Taken together, this indicates a shift in macrophages to a M1 (pro-inflammatory) phenotype and away from a M2 (anti-inflammatory) phenotype in response to a HFD [41]. Identifying the adipose-derived factors that negatively affect tenocytes and macrophages could provide therapeutic pathways to target in the future.

As diet-induced obesity is a modifiable risk factor, reversing obesity through dietary change may be expected to be beneficial for tendon health [7, 8]. We found some evidence to support this with some of the higher gene expression observed in the HFD group not found in the HF-CD group. However, in our first study on this animal cohort investigating tendon healing after rotator cuff surgery, changing from a HFD to CD peri-operatively did not improve healing outcomes [25]. Interestingly, in this study the adipose CM from animals in the HF-CD group had significantly higher expression of pro-inflammatory Il-1β than all other groups. We speculate that higher levels of systemic inflammation from adipose tissue secretions may have contributed to these poor tendon healing outcomes in the HF-CD group. Further research is needed to explore these complex interactions between dietary intervention and tendon health and healing.

This study has several limitations. We used Achilles tendon to analyse the biomechanical and histological effects of a HFD and tail tendon to determine changes in tenocyte gene expression profiles. Ideally, all outcomes would have been explored in the same tendon origin as this would have allowed for correlations to be made between the gene and tissue level changes. Due to practical constraints, we were also limited by the number of tail tendons we could isolate tenocytes from for cellular analyses. This means that we were potentially underpowered for this portion of the study, and it is possible that a larger sample size may have identified further differences. These results should therefore be confirmed with further larger studies.

This animal cohort also underwent upper limb rotator cuff surgery at 15 weeks of age [25], which could have potentially affected the loading and physiology of other tendons throughout the body. However, all animals in the study were similarly affected. Also, only male rats were used in this study, limiting the translatability of our results to both sexes, and in future, it will be important to investigate whether there is a sex-specific response to a HFD. Finally, a longer dietary exposure or older cohort may have resulted in more significant changes in tendon biomechanical and histological properties.

The alterations in tendon cell behaviour and gene expression we observed from exposure to a HFD may predispose tendon to injury and adverse events later in life. There is still an enormous amount of work to be done in understanding the mechanisms and cell signalling pathways linking a HFD, obesity and tendinopathy, in order to determine points for future therapeutic intervention.

## Supplementary Information

Below is the link to the electronic supplementary material.

Supplementary file1 (DOC 51 KB)

Supplementary file2 (DOCX 688 KB)

## Data Availability

This manuscript has associated data in a data repository that is available on request from the corresponding author.

## Abbreviations

ANOVA: Analysis of variance
ARRIVE: Animal research: reporting of in vivo experiments
BMI: Body mass index
CD: Control diet
CM: Conditioned medium
COLIα1: Collagen Iα1
DMEM: F-12: Dulbecco’s modified Eagle’s Medium: Nutrient Mixture F-12
ELISA: Enzyme-linked immunosorbent assay
FBS: Foetal bovine serum
H&E: Haematoxylin–eosin
HF-CD: Dietary intervention group
HFD: High-fat diet
Il: Interleukin
MMP: Matrix metalloproteinase
RPMI: Roswell Park Memorial Institute
SEM: Standard error of the mean
SOX-9: SRY-box-containing gene-9
TEM: Transmission electron microscopy
TNFα: Tumour necrosis factor α
