# Supplementary material for: A high-fat diet has negative effects on tendon resident cells in an in vivo rat model
Source: Int Orthop. 2022 Feb 24;46(5):1181–90. doi: 10.1007/s00264-022-05340-1 (PMC9001221; doi:10.1007/s00264-022-05340-1)
Supplement: Supplementary file 1 — Supplementary file1 (DOC 51 KB) [file 264_2022_5340_MOESM1_ESM.doc]

**The effects of a high-fat diet on tendon: an in vivo rat model**

**International Orthopaedics**

Supplementary methods

Biomechanical testing

The mechanical properties of the Achilles tendon complex were measured using an Instron ElectroPuls E3000 machine (Instron, Norwood, MA, United States), following an established procedure[18, 19]. The tendon width and thickness were measured using digital callipers. Tissue specimens were mounted vertically and subjected to preloading to 0.5N, before a 20-cyle precondition (1 to 20N at a rate of 0.5mm/sec) followed by 1 minute of relaxation. After this, the specimens then stretched to failure at a rate of 0.5mm/sec. Young’s modulus and ultimate load at failure were calculated from the stress-strain curves.

Histological analysis

Excised shoulders were fixed in 10% neutral buffered formalin for 7 days, followed by decalcification in 10% formic acid for 7 days. The samples were transferred to 70% ethanol, processed, embedded in paraffin blocks. Then, 7-μm-thick coronal sections were cut, mounted on slides, stained with haematoxylin-eosin (H&E) and viewed using both transmitted and polarised light.

Cell density (number of nuclei per mm^2^) and nuclear aspect ratio (the ratio of the minor diameter to the maximal diameter, with values approaching zero suggesting a spindle shape and with the value of 1.00 representing a perfect circle) were analysed using the Image J software (NIH, Bethesda, MD, USA), as described in previously established protocols[1, 2]. In healthy tendon, the few tenocytes with flattened nuclei are typically aligned parallel to the tensile axis. In tendinopathy, tendon cells density increases, nuclei become more rounded, and collagen fibre alignment is disrupted[1]. Six regions of interest (ROI) were measured and the mean value was taken for each sample.

The Directionality plug-in for Fiji (http://fiji.sc/Fiji, Ashburn, VA, USA) was used on polarized light images to perform 2D fast Fourier transform analysis to measure collagen fibre alignment, according to previously established methods[3, 4]. The Directionality plug-in calculates the spatial frequencies within an image given a set of radial directions. The method generated normalized histograms revealing the amount of fibres present between 0° and 180° with a bin size of 1°. The plug-in then generates statistics on the highest peak found and performs a Goodness-of-fit test between the observed values and a Gaussian distribution to give a Goodness value (0 (poorly aligned) - 1 (well aligned)). The Goodness values were measured in six ROI’s and the mean value was taken for each sample. Five samples were analysed for each group at 17 and 27 weeks of age.

Collagen fibril diameter measurement with TEM

The Achilles tendon samples were trimmed to only the mid-substance of the tendon and then fixed in 2.5% glutaraldehyde in 0.1M phosphate buffer (pH 7.2) at 4°C overnight and then washed with 0.1M phosphate buffer solution for 10 minutes twice. The samples were then incubated in 1% osmium tetroxide in 0.1M phosphate buffer (pH 7.2) for an hour, washed twice with 2% aqueous sodium acetate for 10 minutes and then twice in distilled water for 10 minutes. En bloc staining was carried out with 2% aqueous uranyl acetate for an hour at 60°C. A series of dehydration steps was carried out in ethanol solutions of 50%, 70%, 80% and 90% ethanol for 10 minutes each and then 100% ethanol for 20 minutes three times. At the end of dehydration process, samples were treated with propylene oxide for 10 minutes twice. Propylene oxide: resin mixtures were prepared in three different ratios of 2:1, 1:1 and 1:2. The samples were incubated in the series of mixtures for an hour each on a rotator and then finally incubated in pure resin for overnight on the rotator, then further incubated for 48 h at 60°C. The resin blocks were thin sectioned (50-70nm) using an ultra-microtome and collected on metal mesh grids (Agar Scientific Ltd, Stansted, UK). Sections were contrasted using uranium acetate and lead citrate before TEM imaging.

The sections were examined using a Tecnai G^2^ spirit twin transmission electron microscope (FEI, Hillsboro, OR, USA) at 120 kV with a Morada camera (Olympus Soft Imaging Solution, Munster, Germany). The diameter of each collagen fibril was analysed using the Image J software. For each sample, at least 5 images were taken from different areas and a total of 25 collagen fibrils were used for analysis from each TEM image. Four samples were analysed for each group at the 17 and 27 week time points.

Primary tenocyte cell culture from tail tendon

Primary rat tenocytes were isolated from tail tendon fascicles, as previously described[5]. Dulbecco’s modified Eagle’s Medium: Nutrient Mixture F-12 (DMEM:F-12), penicillin-streptomycin mixture (10,000 U/mL), and foetal bovine serum (FBS) were obtained from Gibco (ThermoFisher Scientific, Waltham, MA, USA). In brief, tendon fascicles were cut into <1 cm pieces and then placed in 0.5 mg/ml dispase and 0.5 mg/ml collagenase (both from Sigma-Aldrich) in DMEM: F-12 with 10% FBS at 37°C for up to 18-h until most of the extra cellular matrix had been digested.  The cell suspension was then passed through a cell strainer, washed and re-suspended in enzyme-free media. Cells were cultured in DMEM: F- 12 with 10% FBS in 75 cm^2^ flasks (Corning Inc., Corning, NY, USA) and incubated at 37°C with 5% CO_2_ until confluent. Three samples were analysed for each group and each time point.

## **Tenocyte growth assays**

Primary rat tenocytes from each group were seeded in 24-well plates (Greiner BioOne, Kremsmünster, Austria), at a density of 2.5×10^4^ cells/well and cultured in DMEM: F-12 with 5% FBS. Following 24-h, cell growth was measured again using alamarBlue (Life Technologies, ThermoFisher Scientific Inc.) at 5% of final concentration in well for 4-h at 37°C. Following this incubation, 200μl of the alamarBlue conditioned medium (CM) was transferred to a 96-well plate (Greiner Bio-One) and fluorescence (excitation 540nm; emission 630nm) was read using a Synergy 2 multi-detection microplate reader (BioTek Instruments Inc., Winooski, VT, USA). Fluorescent intensity is a linear measure of cell numbers[6]. Each well was changed to fresh media and the process to analyse cell growth was repeated at 48-h and 72-h. There were 4 wells per biological repeat, with 3 biological repeats per group and time point.

## **Tenocyte collagen deposition**

Primary rat tenocytes were seeded in 24-well plates at a density of 7.5×10^4^ cells/well and incubated in DMEM: F-12 with 5% FBS and 50 μg/ml L-ascorbic acid 2-phosphate. After 72 h culture, cells were fixed with Bouin’s solution (71% saturated picric acid, 24% formalin, 5% 0.5M acetic acid) for 30 minutes and then stained with 0.1% Sirius red dissolved in saturated picric acid for 1 h. At the end of this incubation, cells were washed with 0.01M hydrochloric acid five times and left to air dry. The dye was released using 0.1M sodium hydroxide and 200μl of the released dye was transferred to a 96-well plate. Absorbance was measured at 570nm using a Synergy 2 multi-detection microplate reader. There were 4 wells per biological repeat, with 3 biological repeats per group and time point.

## **Adipose tissue conditioned media tenocyte culture**

Adipose CM was used to study the depot-specific effect of visceral omental fat on rat tenocytes. CM was generated by incubating the adipose tissue explants in a standardized volume of DMEM:F-12 with 1% FBS (200mg tissue: mL media) in a 75cm^2^ flask. The CM was collected at 48-h, filter-sterilized and stored at −80 °C until use. Adipose tissue samples were collected from each group of animals at the 27 week time point.

Tenocytes obtained from tail tendon of a 17-week-old male rat fed a control diet (CD) were then cultured in adipose CM and DMEM:F12 with 1% FBS (ratio 3.5:6.5) for 72-h. Cell growth assays with alamarBlue and collagen deposition assays with Sirius red were then performed as described above, except readings were taken after 48-h cell growth.

Adipose tissue conditioned media THP-1 cell culture

Human monocytic THP-1 cells were sourced from the American Tissue Culture Collection (TIB-202™, ATCC, Manassas, VA, USA). THP-1 cells were cultured in 75cm^2^ flasks with Roswell Park Memorial Institute (RPMI) cell media (Invitrogen™, ThermoFisher Scientific, Australia) supplemented with 10% FBS and incubated at 37°C with 5% CO_2_ until the required number of cells were present. Cells were then seeded in 24-well plates at a density of 1.5×10^6^ cells/well in RPMI media supplemented with 10% FBS, and activated to macrophage-like cells with the addition of 200 µg/mL phorbol myristate acetate. After 24-h, media was removed and replaced with adipose CM and RPMI with 10% FBS (ratio 3.5:6.5) from each group or DMEM: F12 and RMPI with 10% FBS for the control media. Plates were incubated for 48-h post-seeding. Cell pellets were then harvested, washed and stored at -80 °C for a period before being used for gene expression analysis.

Cytokine profiling in adipose tissue conditioned media

The concentration of adiponectin and leptin in the adipose CM were analysed by commercial rat-specific enzyme-linked immunosorbent assays (ELISA; Crystal Chem, Chicago, IL, USA). Insulin, interleukin (Il)-1β, Il-6, and tumour necrosis factor α (TNFα) were analysed by Quantikine ELISA (R&D Systems; Minneapolis, MN, USA).

## **Gene expression analysis**

For analysis of gene expression, total cellular RNA was extracted from cultured cells and purified using the RNeasy minikit (Qiagen, Venlo, The Netherlands). Genomic DNA was removed using RNasefree DNase set (Qiagen). Purity and concentration of the extracted RNA was measured using NanoDrop Lite Spectrometer (Thermo-Fisher, Victoria, Australia). Complementary-DNA (cDNA) was prepared by using 500ng of RNA with super-script-III (Life Technologies, Carlsbad, CA, USA). Primer-probe sets were purchased as TaqMan Gene Expression Assays (Life technologies). Multiplex quantitative polymerase chain reaction (qPCR) was performed with FAM specific for genes of interest and VIC-labelled 18S endogenous ribosomal RNA probes, according to the manufacturer’s instructions, using an ABI PRISM 7900HT sequence detection system (Applied Biosystems, Foster City, CA, USA). Samples were assayed in triplicate. The ΔΔCt calculation method was used to determine the relative level of messenger RNA expression[7], normalized to the values of cells from the tail tendon of a 17-week-old male rat fed a CD. For tenocytes, the relative gene expression of collagen Iα1 (COLIα1), collagen IIIα1 (COLIIIα1), cyclooxygenase-2 (COX-2), scleraxis, SRY-box containing gene-9 (SOX-9), tendomodulin, matrix metalloproteinase (MMP) -3 and MMP-13 were determined. For THP-1 cells, the relative gene expression of pro-inflammatory cytokines Il-1β, Il-8, TNFα and anti-inflammatory cytokine Il-10 were determined.

Abbreviations

CD: control diet

COLIα1: collagen Iα1

COLIIIα1: collagen IIIα1

COX-2: cyclooxygenase-2

cDNA: complementary-DNA

DMEM:F-12: Dulbecco’s modified Eagle’s Medium: Nutrient Mixture F-12

ELISA: enzyme-linked immunosorbent assay

FBS: foetal bovine serum

Il: interleukin

MMP: metalloproteinase

qPCR: quantitative polymerase chain reaction

ROI: regions of Interest

RPMI: Roswell Park Memorial Institute

SOX-9: SRY-box containing gene-9

TEM: transmission electron microscopy

TNFα: tumour necrosis factor α

References

1. Andŕes JFS, Domínguez JM, Granados MM, Morgaz J, Navarrete R, Carrillo JM, Ǵomez-Villamandos RJ, Mũnoz-Rasćon P, De Las Mulas JM, Milĺan Y, García-Balletbó M, Cugat R (2013) Histological study of the influence of plasma rich in growth factors (PRGF) on the healing of divided achilles tendons in sheep. J Bone Jt Surg - Ser A 95:246–255 . https://doi.org/10.2106/JBJS.K.01659

2. Rooney SI, Baskin R, Torino DJ, Vafa RP, Khandekar PS, Kuntz AF, Soslowsky LJ (2016) Ibuprofen differentially affects supraspinatus muscle and tendon adaptations to exercise in a rat model. Am J Sports Med 44:2237–2245 . https://doi.org/10.1177/0363546516646377

3. Sensini A, Gualandi C, Cristofolini L, Tozzi G, Dicarlo M, Teti G, Mattioli-Belmonte M, Letizia Focarete M (2017) Biofabrication of bundles of poly(lactic acid)-collagen blends mimicking the fascicles of the human Achille tendon. Biofabrication 9: . https://doi.org/10.1088/1758-5090/aa6204

4. Sensini A, Gualandi C, Zucchelli A, Boyle LA, Kao AP, Reilly GC, Tozzi G, Cristofolini L, Focarete ML (2018) Tendon Fascicle-Inspired Nanofibrous Scaffold of Polylactic acid/Collagen with Enhanced 3D-Structure and Biomechanical Properties. Sci Rep 8: . https://doi.org/10.1038/s41598-018-35536-8

5. Musson DS, Tay ML, Chhana A, Pool B, Coleman B, Naot D, Cornish J (2017) Lactoferrin and parathyroid hormone are not harmful to primary tenocytes in vitro, but PDGF may be. Muscles Ligaments Tendons J 7:215–222 . https://doi.org/10.11138/mltj/2017.7.2.215

6. Back SA, Khan R, Gan X, Rosenberg PA, Volpe JJ (1999) A new Alamar Blue viability assay to rapidly quantify oligodendrocyte death. J Neurosci Methods 91:47–54 . https://doi.org/10.1016/S0165-0270(99)00062-X

7. Livak KJ, Schmittgen TD (2001) Analysis of relative gene expression data using real-time quantitative PCR and the 2-ΔΔCT method. Methods 25:402–408 . https://doi.org/10.1006/meth.2001.1262
