# Supplementary material for: A high-fat diet has negative effects on tendon resident cells in an in vivo rat model
Source: Int Orthop. 2022 Feb 24;46(5):1181–90. doi: 10.1007/s00264-022-05340-1 (PMC9001221; doi:10.1007/s00264-022-05340-1)
Supplement: Supplementary file 2 — Supplementary file2 (DOCX 688 KB) [file 264_2022_5340_MOESM2_ESM.docx]

**The effects of a high-fat diet on tendon biomechanical properties, structure and gene expression in a rat model**

**International Orthopaedics**

**Supplementary Figures and Tables**

**Supplementary Figure 1.** Averaged directionality histograms of collagen fibre alignment for each group. Averaged histograms demonstrating the distribution of collagen fibres present between 0° and 180° with a bin size of 1° for each time point and dietary group. n=5 per group.

**
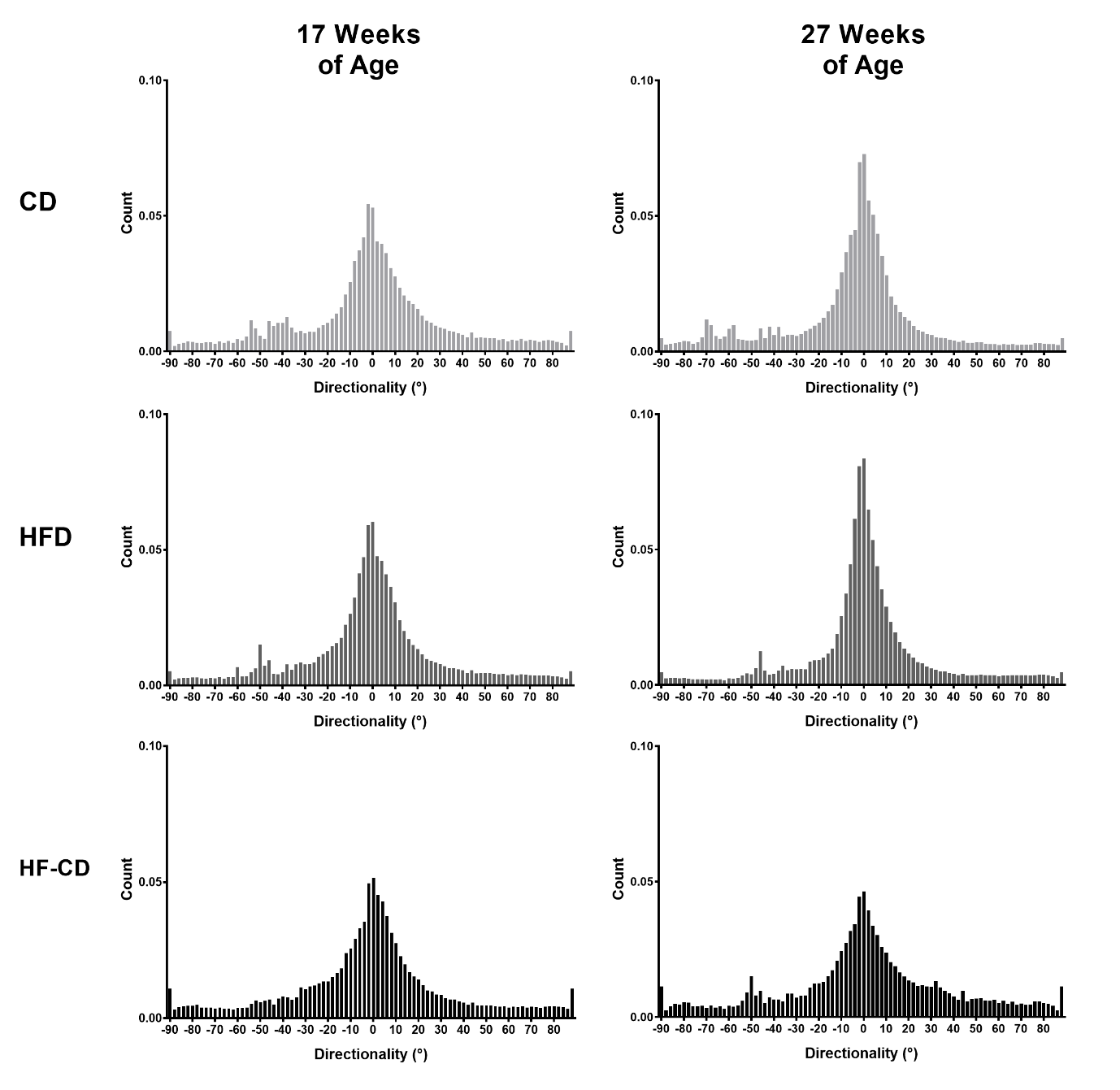
**

**Supplementary Figure 2.** Collagen fibril diameter of the mid-substance of Achilles tendon. Data are presented as mean ± SEM (n=4 per group)


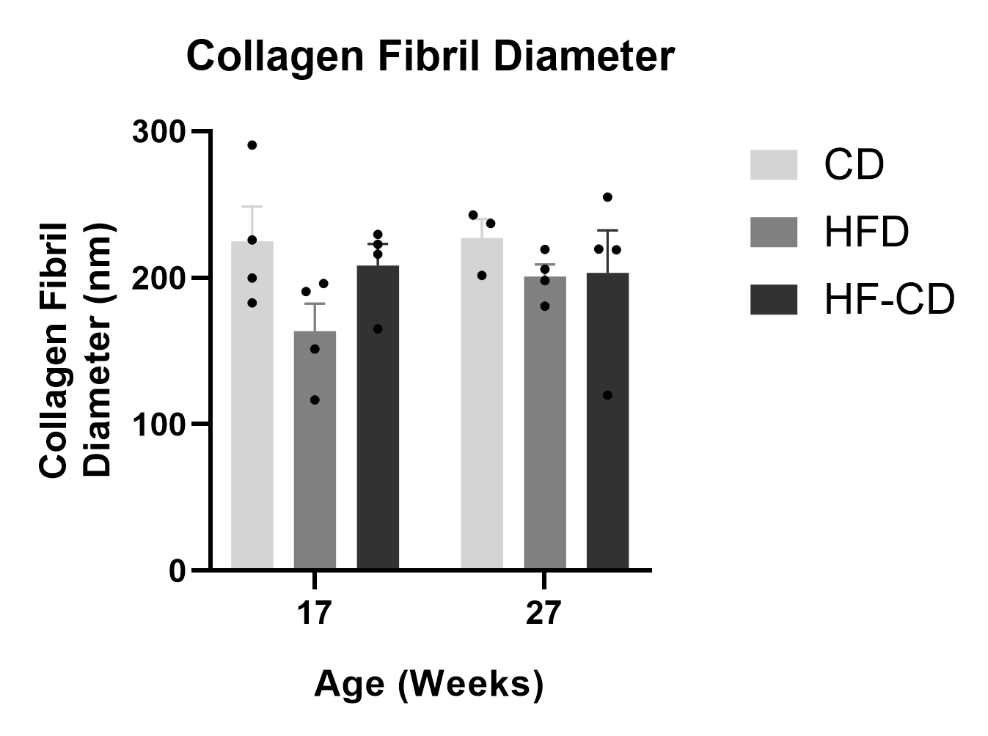


**Supplementary Figure 3.** Frequency distribution of collagen fibrils measured for each group. Collagen fibrils in Achilles tendons were grouped according to diameter, showing a unimodal distribution (n=4 per group).

**
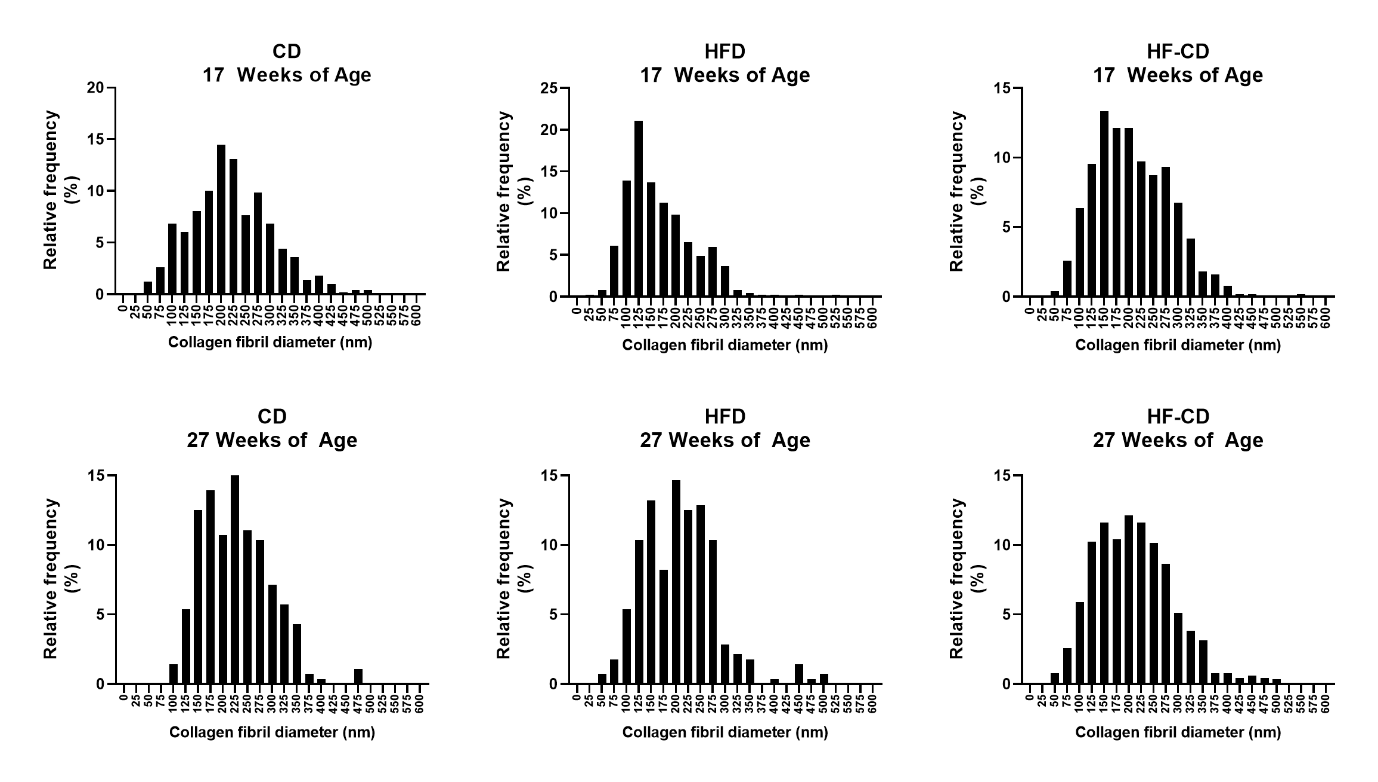
**
